# Supplementary material for: The Geriatric Nutritional Risk Index Predicts Prognosis in Japanese Patients with LATITUDE High-Risk Metastatic Hormone-Sensitive Prostate Cancer: A Multi-Center Study
Source: Cancers (Basel). 2023 Nov 8;15(22):5333. doi: 10.3390/cancers15225333 (PMC10670086; doi:10.3390/cancers15225333)
Supplement: Supplementary file 1 [file cancers-15-05333-s001.zip › cancers-2694880-supplementary.pdf]

Supplementary Materials: The Geriatric Nutritional Risk Index Predicts Prognosis in Japanese Patients with LATITUDE High-Risk Metastatic Hormone-Sensitive Prostate Cancer: A Multi-Center Study

Taku Naiki, Kiyoshi Takahara, Hiromitsu Watanabe, Keita Nakane, Yosuke Sugiyama, Takuya Koie, Ryoichi Shiroki, Hideaki Miyake and Takahiro Yasui

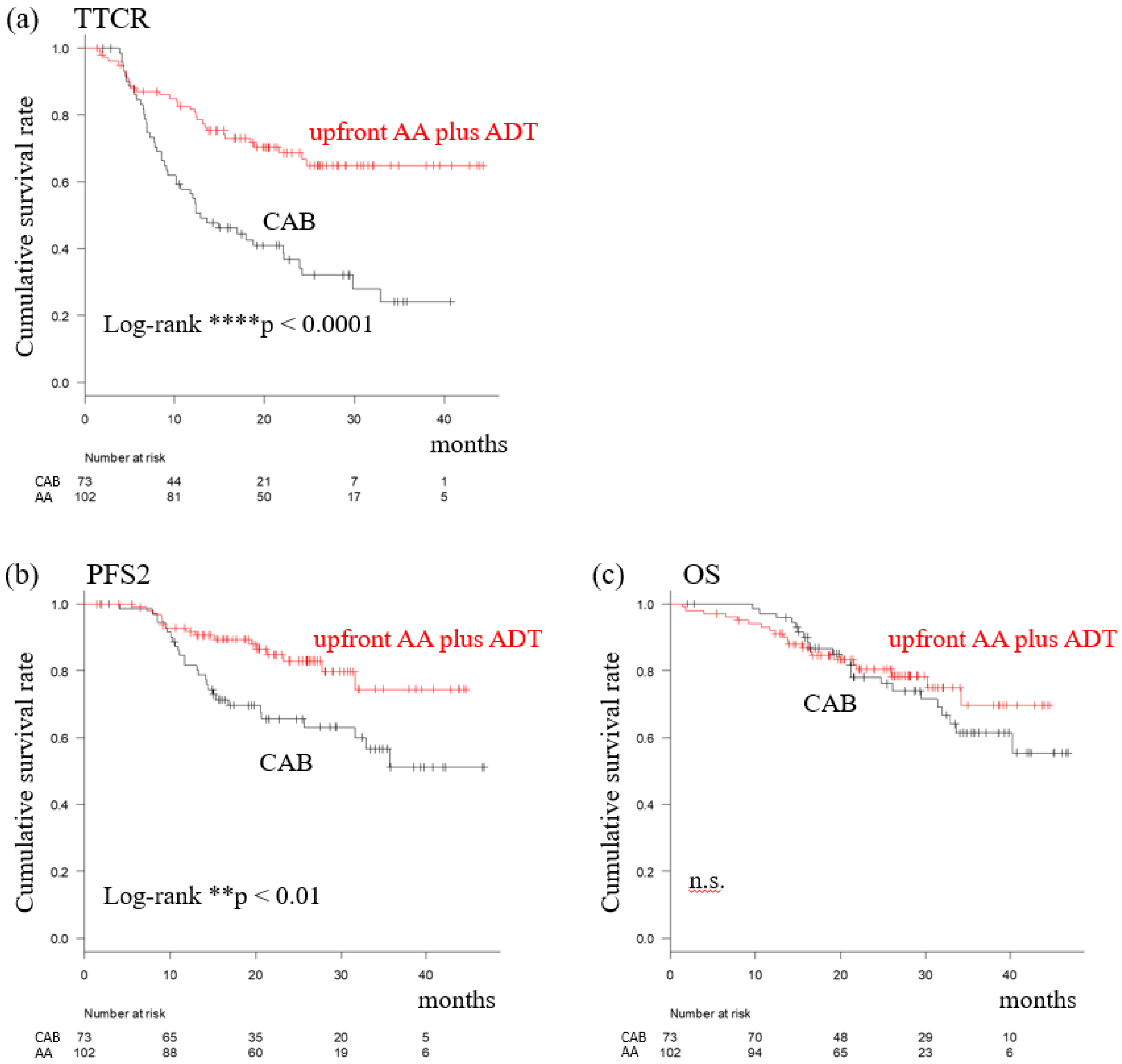

Figure S1. TTCR (**a**), PFS 2.

Table S1. Patients' characteristics and oncological outcomes for CAB and upfront AA plus ADT groups.

Characteristics		CAB group (n=73)	Upfront AA plus ADT group (n=102)	*p* value
**Median age, years (range)**		76 (58–91)	74 (48–87)	0.16
**Median initial serum PSA levels, ng/ml (range)**		227 (3.5–1000)	265 (0.8–16720)	0.15
**Median BMI, kg/m² (range)**		22.7 (16.3–34.1)	22.6 (14.2–31.6)	0.31
**Gleason score at initial diagnosis, n (%)**	**4+3**	1 (1.4)	1 (1.0)	0.15
	**4+4**	35 (47.9)	32 (31.4)	
	**4+5**	21 (28.8)	36 (35.3)	
	**5+4**	12 (16.4)	20 (19.6)	
	**5+5**	4 (5.5)	13 (12.7)	
**Clinical T stage**	**2a**	0 (0)	4 (3.9)	0.36
	**2b**	5 (6.8)	3 (2.9)	
	**2c**	5 (6.8)	11 (10.8)	
	**3a**	15 (20.5)	25 (24.5)	
	**3b**	25 (34.2)	29 (28.4)	
	**4**	23 (31.5)	30 (29.4)	
**Clinical N stage**	**N0**	40 (54.8)	47 (46.1)	0.53
	**N1**	32 (43.8)	52 (51.0)	
	**N2**	1 (1.4)	3 (2.9)	
**Visceral metastasis, n (%)**	**no**	52 (71.2)	65 (63.7)	0.33
	**yes**	21 (28.8)	37 (36.3)	
**ECOG–PS, n (%)**	**0**	50 (68.5)	77 (75.5)	0.31
	**1**	17 (23.3)	13 (12.7)	
	**2**	5 (6.8)	10 (9.8)	
	**3**	1 (1.4)	2 (2.0)	
**Existence of symptom, n (%)**		42 (57.5)	60 (58.8)	0.89
**The use of bone modifying agents, n (%)**		38 (52.1)	64 (62.7)	0.17
**Median serum albumin level, g/dl (range)**		3.9 (1.5–6.6)	3.9 (2.2–4.8)	0.76
**Median serum ALP level, U/l (range)**		372 (81–8300)	446 (139–11620)	0.55
**GNRI level**	**low**	14 (19.2)	22 (21.6)	0.85
	**high**	59 (80.8)	80 (78.4)	
**50% decline in PSA, n (%)**		61 (83.6)	88 (86.3)	0.67
**Median period to PSA nadir, days (range)**		201 (37–980)	298 (21–1215)	0.13
**CRPC development, n (%)**		47 (64.4)	31 (30.4)	< 0.001***

AA: Abiraterone acetate, ADT: Androgen deprivation therapy, ALP: Alkaline phosphatase, Bica: bicalutamide, BMI: Body mass index, CAB: Combined androgen blockade, CRPC: Castration-resistant prostate cancer, ECOG–PS: Eastern Cooperative Oncology Group performance status, GNRI: Geriatric nutritional index, PSA: Prostate specific antigen.
